# Symptom profile in suicide attempters during the COVID-19 pandemic: Relationships with suicide outcomes

**DOI:** 10.1192/j.eurpsy.2024.1759

**Published:** 2024-10-25

**Authors:** Patricia Díaz-Carracedo, Carolina Marín, Marina Diaz-Marsa, Guilherme Borges, Alejandro de la Torre-Luque, Matilde Elices, Alba Toll, Iria Grande, Natalia Roberto, Mireia Vázquez, Ana González-Pinto, Miguel Ruiz-Veguilla, Manuel Canal-Rivero, Ana I. Cebria, Diego Palao, Teresa Bobes-Bascaran, Luis Jimenez-Treviño, Pilar Saiz, Jorge Andreo-Jover, Katya March, Angela Palao-Tarrero, The SURVIVE Collaborative Signature, Víctor Perez

**Affiliations:** 1 Department of Legal Medicine, Psychiatry and Pathology. School of Medicine, Complutense University of Madrid, Madrid, Spain; 2 Instituto Nacional de Psiquiatria Ramon de la Fuente Muniz, Mexico City, Mexico; 3 Centro de Investigacion Biomedica en Red de Salud Mental (CIBERSAM), Madrid, Spain; 4 Hospital Clinic de Barcelona, Barcelona, Spain; 5 Virgen del Rocio University Hospital, Seville, Spain; 6 Parc Tauli Foundation – UAB University Institute, Barcelona, Spain; 7 La Paz University Hospital, Madrid, Spain; 8 Universitat de Barcelona, Barcelona, Spain; 9 Araba University Hospital, Araba, Spain; 10 University of Oviedo, Oviedo, Spain; 11 Autonomous University of Madrid, Madrid, Spain; 12 Hospital del Mar Institute for Medical Research, Barcelona, Spain

**Keywords:** medical injury, psychopathology symptoms, suicidal ideation, suicide attempt, symptom profile

## Abstract

**Background:**

Suicidal behavior constitutes a multi-cause phenomenon that may also be present in people without a mental disorder. This study aims to analyze suicidal behavior outcomes in a sample of attempters, from a symptom-based approach.

**Methods:**

The sample comprised 673 patients (72% female; *M* = 40.9 years) who attended a hospital emergency department due to a suicide attempt. A wide range of clinical factors (e.g., psychopathology symptoms, psychiatric diagnoses, impulsivity, acquired capability), was administered within 15 days after the index attempt. Nine psychopathology domains were explored to identify the profile of symptoms, using latent profile analysis. The relationship between the profile membership and suicide outcome (i.e., intensity of suicidal ideation, number of suicide behaviors, and medical injury derived from index attempt) was also studied, using linear and logistic regression.

**Results:**

Three psychopathology profiles were identified: high-symptom profile (45.02% of participants), moderate-symptom profile (42.50%), and low-symptom profile (12.48%). High-symptom profile members were more likely to show higher risk of non-suicidal self-injury, acquired capability for suicide, and more severe suicide behavior and ideation. On the other hand, a more severe physical injury was associated with low-symptom profile membership in comparison to membership from the other profiles (*OR* < 0.45, *p* < .05).

**Conclusions:**

A symptom-based approach may be useful to monitor patients and determine the risk of attempt repetition in the future and potential medical injury, and to optimize prevention and intervention strategies.

## Introduction

More than 700,000 people took their own lives in 2019. In other words, suicide was the cause of 9 deaths per 100,000 inhabitants, worldwide [1]. The impact of suicide remains dramatic beyond young age, as it is one of the main causes of preventable, non-natural death, and may affect all age groups, geographic regions, and all socioeconomic statuses. Suicide-related behavior (SRB), particularly suicide attempt and reattempt, constitutes a critical risk factor of death by suicide. In this regard, it is estimated that there may be 25 attempts for one death by suicide [2, 3].

Among the key factors for SRB, a particular interest is put on psychiatric conditions and mental health. More concretely, some seminal studies have stated that between 70 and 90% of people who died by suicide had shown the diagnosis of a mental disorder [4–6]. Additionally, patients with a mental disorder may show three times higher risk of engaging in either a suicide attempt or attempt repetition, in comparison to people without a diagnosis [7–9]. Finally, co-occurrence of several disorders may substantially increase the risk of suicide [10, 11], by means of influencing on risk factors of SRB, such as the acquired capability for suicide [12, 13].

From a dimensional standpoint, a mental disorder should be considered along the continuum of disease progression, from normal functioning to a full-blown disorder [14, 15]. Subthreshold disorders (depicting lower levels of severity and interference) or the so-called at-risk stages are therefore considered intermediate stages, falling along the same continuum, qualitatively similar but quantitatively less severe. Mounting evidence has shown a clear relationship between SRB and prodromal conditions featured by alterations in key neurophysiological axes (e.g., hypothalamic- pituitary–adrenal axis), and subclinical disorders [16–18].

Comorbidity patterns of psychopathology symptoms may be involved in the configuration of a suicidal attempt and its main features (i.e., related ideation, self-injury presence, and physical injury). Previous studies have identified patterns of symptom comorbidity among suicide attempters [19, 20]. Unfortunately, these studies were focused on young samples, overlooking the distinctive features of suicide behavior across the lifespan [21, 22]. This study aimed to expand the findings provided by previous studies in terms of identifying the symptom profiles of adult attempters who were admitted to a hospital emergency department due to a suicide attempt. We expect to find at least two different profiles of psychopathology symptoms in our sample of attempters, taking into account that no clear evidence exists in terms of symptom dynamics in people with active suicide behavior. Moreover, it intended to study the relationship between the symptom profile membership and risk factors (i.e., acquired capability for suicide, impulsivity, self-injury), for suicide and outcomes (i.e., presence and intensity of suicidal ideation, presence and number of suicide-related behaviors and injury severity of current suicide attempt). We expect that attempters with a profile featured by high levels of symptoms across psychopathology domains would show higher risk across suicide outcomes. This may constitute an outstanding window of opportunity to take more accurate evidence on the pandemic and anti-COVID measures on suicide outcomes. Thus, it is necessary to take into account the social context, worldwide, and its possible impact on disease progression and daily functioning.

## Methods

### Participants

Data from this study come from the SURVIVE study [23]. Participants aged 18 years and older were recruited at the psychiatric emergency ward of eight public, general, university hospitals across Spain (i.e., Hospital Clinic Barcelona, Corporació Sanitària Parc Taulí, Hospital del Mar, Hospital Clínico San Carlos, Hospital Universitario La Paz, Hospital Universitario Araba-Santiago, Hospital Universitario Virgen del Rocio, Hospital Universitario Central de Asturias). The sample comprised 673 patients (72% female; *M* age = 40.9, *SD* = 15.2). Sample was recruited between November/2020 and January/2022, during the COVID-19 times. Patients provided a written, informed consent to participate in this study. All the protocols conducted within the SURVIVE study were approved by an Ethical Committee for Human Research from each of the recruiting sites.

### Data collection

The participants were assessed for a trained mental health professional (i.e., clinical psychologist or psychiatrists) using a wide battery of clinical tools, within the 15 days after the emergency department admission. First, a sociodemographic interview was administered to assess sociodemographic factors (e.g., sex, age, employment status, marital status). In addition, the Mini-International Neuropsychiatric Interview (M.I.N.I.) [24], version 7.0.2, was used as a clinical interview based on the Diagnostic and Statistical Manual for Mental Disorders (DSM-5) [25], to ascertain the presence of psychiatric disorders.

Some additional tools were delivered: the Brief Symptom Inventory (BSI) [26] to assess the psychopathology symptoms according to nine domains (i.e., anxiety, depression, hostility, OCD, paranoid, phobic, psychoticism, sensitivity and somatization); the Barratt Impulsiveness Scale (BIS-11) [27] to measure impulsivity levels; the Acquired Capability for Suicide Scale-Fearlessness about Death (ACSS-FAD [28] to measure fearlessness about death and pain tolerance; and the Columbia-Suicide Severity Rating Scale (C-SSRS) [29] to assess suicidal behavior.

### Data analysis

Latent profile analysis (LPA) was used to enumerate the profile of symptoms according to the nine BSI symptom domains. Under the Gaussian Finite Mixture Modeling tradition, LPA assumes that there may be a latent factor (i.e., symptom profile) leading to the patterns of responses on a series of indicator items [30, 31]. LPA is based on a comparison approach by which solutions with an increasing number of symptom profile classes are compared. A solution with a better fit to data is supported by: (1) lower levels of both the Bayesian information criterion (BIC) and the integrated complete-data likelihood (ICL) indexes; (2) significant *χ* ^2^ statistic derived from the bootstrap likelihood ratio test (BLRT); and (3) more than 5% of sample within every identified class.

Pearson’s *χ* ^2^ test (categorical data) and analysis of variance *F*-based test (continuous data) were conducted to explore significant differences between symptom profiles in terms of sociodemographic and clinical profiles. Finally, generalized linear modeling (GLM) was followed to study the relationship between the suicide outcomes and the symptom profile membership, as well as other sociodemographic (i.e., sex at birth, age, nationality, marital status, educational attainment, working status) and clinical factors (i.e., number of psychiatric diagnoses, acquired capability for suicide, presence of non-suicidal self-injury, impulsivity and number of previous suicide attempts). More concretely, logistic binary regression was used for the presence of ideation, the presence of suicide behaviors, and the severity of current attempt outcomes; whereas GLM assuming a gamma distribution for the outcome was used for the ideation intensity and the number of suicide behaviors outcomes. The Akaike information criterion (AIC) was used to compare the model with covariates and the other without covariates (i.e., unconstrained model), to visualize the contribution of the covariates on each outcome variance; as well as the adjusted Nagelkerke’s pseudo-*R* ^2^ as an effect size estimate of the whole model. The odds ratio (*OR*) estimate was used to see the magnitude of the association between each covariate and the outcome.

Analyses were conducted using the R software (mclust, psych, rsq packages).

## Results

Table 1 displays the sociodemographic and clinical features of the sample in analysis. A total of 673 adults were included. Most participants were women (71.6%) with a mean age of 40.92 (*SD* = 15.5) years old, with at least two comorbid psychiatric disorders on average and high levels of impulsivity. More than 4 in 10 participants reported having engaged in self-injury, with at least three previous suicide attempts on overall. Regarding suicide-related outcomes, more than 90% of the sample reported suicidal ideation and 70% with suicide-related behavior, being preparatory acts the most prevalent suicide-related behavior (38.19% of patients). Finally, 13% of patients showed attempts with severe medical injury. The most common suicide method was self-poisoning with solid or liquid substances (82.2%). The most prevalent mental disorders were major depressive disorder (59.3% of participants), panic disorder (20.8%), alcohol use disorder (16.8%), and generalized anxiety disorder (34%).

**Table 1.** Sociodemographic and clinical features of sample

Sample feature	Statistic
Sex (%female)	71.62
Age	40.92 (15.5)
Nationality (%foreign-born)	22.59
Marital status (%married/coupled)	40.56
Educational attainment	
Primary education	22.44
Secondary education	51.11
Tertiary education	26.45
Working status	
Unemployed/retired	52.92
Active	36.43
Student	10.64
BSI	
GSI	1.85 (0.81)
PST	37.26 (11.39)
PSDI	2.55 (0.65)
Number of diagnoses	2.08 (1.79)
Impulsivity^a^	74.63 (5.80)
Acquired capability^b^	18.17 (6.75)
Non-suicidal self-injury (%yes)	41.16
Number of previous attempts	3.45 (5.62)
Presence of suicidal ideation (%yes)	91.83
Suicidal ideation intensity	18.98 (7.84)
Presence of SRB (%yes)	70
Number of SRB	1.26 (1.09)
Current suicide attempt	
Medical damage (%severe)	13.08
Method	
Poisoning with solid or liquid substances	82.17
Poisoning with gases or vapors	1.63
Stabbing	6.98
Drowning	0.15
Savings or strangulation	2.82
Jumping from high buildings	1.78
Others	4.46

*Note:* The percentage of cases is displayed for dichotomous and categorical variables. Mean and standard deviation (between brackets) are displayed for continuous variables.

Abbreviations: BSI, Brief Symptom Inventory; GSI, Global Severity Index; PSDI, Positive Symptom Distress Index; PST, positive symptoms total; SRB, suicide-related behavior.

a Total score of the Barratt Impulsiveness Scale (BIS-11).

b Total score from the Acquired Capability for Suicide Scale (ACSS).

**Figure 1.**
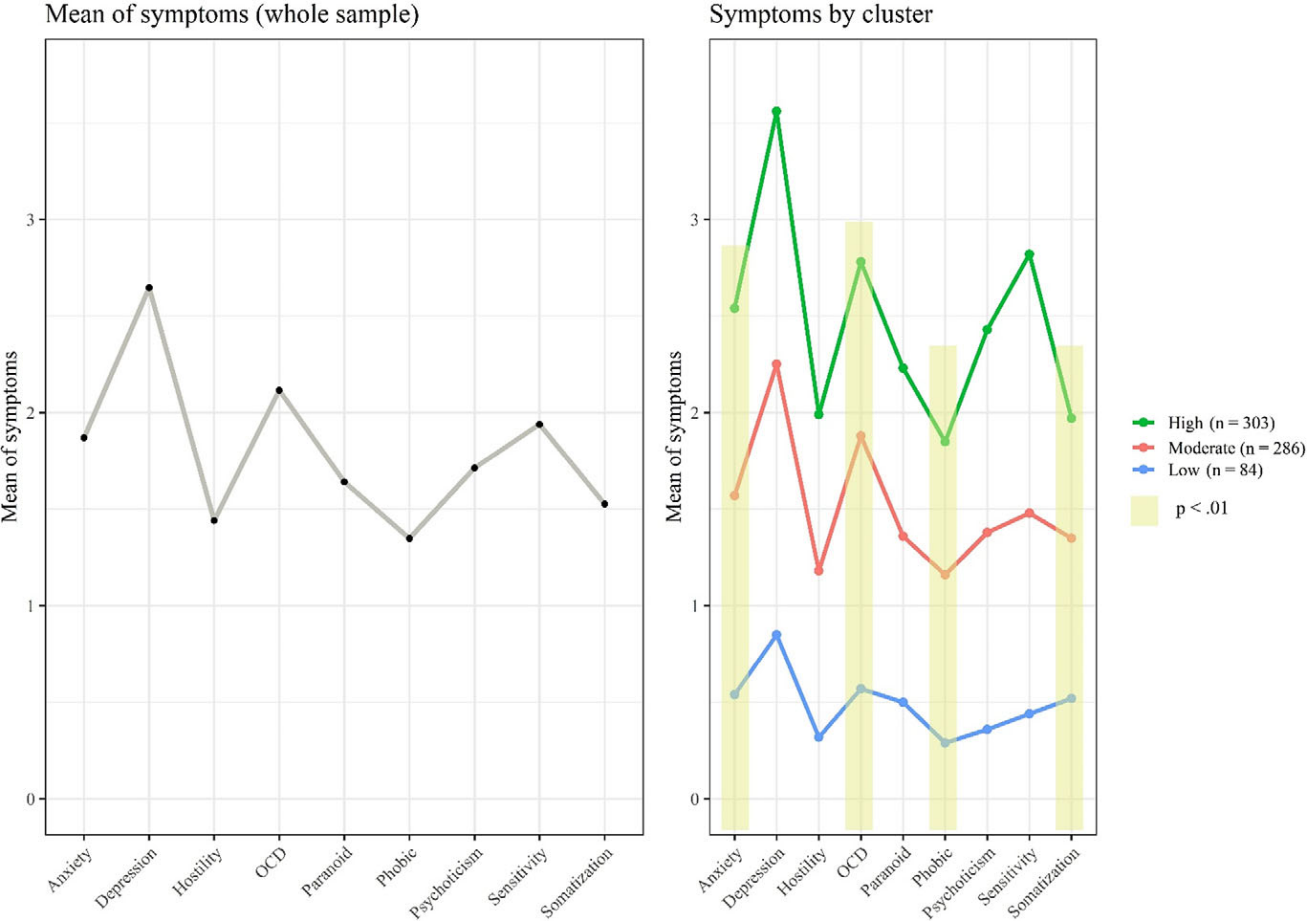
Symptom dimensions across the symptom profile classes. The overall level of symptoms for the whole sample is displayed on the left box. The overall level of symptoms according to symptom profile class is displayed on the right box. The symptom dimensions are derived from the Brief Symptom Inventory. The yellow-shaded area reflects dimensions with significant differences between groups, with *p* < .01. OCD, obsessive-compulsive disorder symptoms.

With regard to symptom profile identification, the LPA revealed that the model with three classes fitted better to data (BIC = −13466.96, ICL = −13584.02, BLRT = 180.21, *p* < .01). The fit indexes for the LPA solutions are displayed in Table 2. Regarding the identified classes, a class (so-called low-symptom class) comprising 12.48% was identified. This class showed a minimal level across symptom dimensions (see Figure 1). The moderate-symptom class (42.50% of participants) was identified featured by moderate levels of symptoms across dimensions. Finally, the high-symptom class (45.02% of participants) was identified. Participants from this class showed the highest levels of symptoms across dimensions (Figure 1). Significant differences were found between classes in terms of four symptom domains: Anxiety, *F* (1, 671) = 4.38, *p* < .05, *η* ^2^_partial_ = 0.01; OCD, *F* (1, 671) = 9.75, *p* < .01, *η* ^2^_partial_ = 0.01; Phobic, *F* (1, 671) = 6.47, *p* < .05, *η* ^2^_partial_ = 0.01; and Somatization, *F* (1, 671) = 6.66, *p* < .05, *η* ^2^_partial_ = 0.01. Pairwise comparison under the Bonferroni correction pointed to significant differences between the three groups in the aforementioned symptom dimensions (*p* < .01, for all the comparisons).

**Table 2.** Latent profile analysis solutions

Class	LL	BIC	ICL	BLRT	%n in class
1	−6722.95	−13797.53	−13797.53		100
2	−6523.46	−13493.1	−13550.93	398.97*	13.56–85.44
3	−6433.56	−13466.96	−13584.02	180.21*	12.48–45.02
4	−6391.75	−13469.7	−13714.77	83.21*	11.59–37.59
5	−6347.24	−13524.89	−13721.08	89.03*	3.86–38.63
6	−6299.39	−13515.43	−13801.6	95.70*	4.46-–28.38
7	−6259.12	−13504.11	−13852.73	80.53*	4.75–29.42
8	−6224.22	−13525.74	−13666.2	69.80*	4.61–24.67
9	−6159.61	−13567.01	−13908.89	129.23*	2.53–21.10

*Note:* ‘Class’ refers to number of classes considered in each model. ‘%n in class’ refers to percentage of participants in each class.

Abbreviations: BIC, Bayesian information criterion; BLRT, Bootstrap likelihood ratio test; ICL, integrated complete-data likelihood criterion; LL, maximum log-likelihood estimator for model convergence.

* *p* < .01.

**Figure 2.**
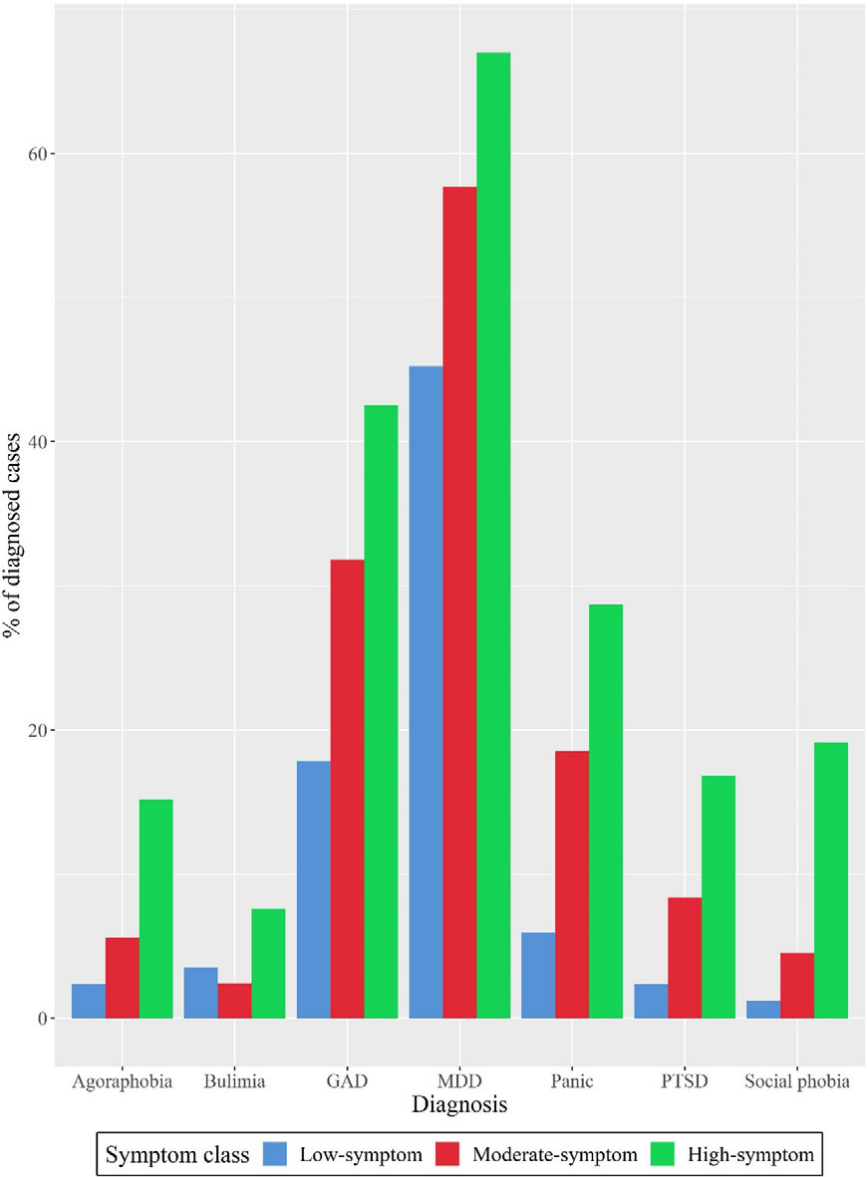
Psychiatric diagnoses with significant differences between symptom classes. The diagnoses are derived from the Mini International Neuropsychiatric Interview delivery. GAD, generalized anxiety disorder; MDD, major depressive disorder; PTSD, posttraumatic stress disorder.

Class membership was also related with the BSI summary indexes, observing higher values in the high-symptom class, in comparison to the other classes (with higher levels in the moderate-symptom class than the low-symptom class). Sociodemographic and clinical features according to symptom class are displayed in Table 3. Participants from the low-symptom class were more likely to be women, older, and unemployed/retired (*p* < .01 for all these factors). Regarding clinical factors, participants from the high-symptom profile showed more diagnoses than the remaining classes, as well as higher acquired capability for suicide. No significant differences were shown between classes in terms of impulsivity. Regarding specific psychiatric disorders, significantly higher proportion of high-symptom participants was observed for major depressive disorder several anxiety disorders (i.e., panic disorder, agoraphobia, social phobia, and generalized anxiety), posttraumatic stress disorder, and bulimia nervosa (*p* < .01, for all the comparisons). Figure 2 displays the proportion of participants with the aforementioned diagnoses according to symptom class. Finally, higher levels of suicidal ideation and SRB were found in participants from the high-symptom class. Conversely, a higher proportion of participants from the low-symptom class showed severe medical injury in comparison to the other groups, *χ* ^2^(2) = 10.27, *p* < .01, Cramer’s *V* = 0.08. No between-class difference was observed in terms of the current attempt method (see Table 3).

**Table 3.** Sociodemographic and clinical features according to symptom profile cluster

	Low-symptom profile	Moderate-symptom profile	High-symptom profile	Contrast test	ES
Sex (%female)	55.95	71.33	76.24	13.34**	0.14
Age	49.3 (16.75)	42.89 (15.43)	36.75 (13.86)	27.58**	0.08
Nationality (%foreign-born)	16.67	20.98	25.74	3.83	0.05
Marital status (%married/coupled)	46.43	43.71	35.97	5.02	0.06
Educational attainment				5.02	0.06
Primary education	22.62	23.43	21.45		
Secondary education	52.38	50.7	51.16		
Tertiary education	25	25.87	27.39		
Working status				24.22**	0.11
Unemployed/retired	64.29	55.09	47.65		
Active	32.14	38.6	35.57		
Student	3.57	6.32	16.78		
BSI					
GSI	0.52 (0.24)	1.58 (0.48)	2.47 (0.52)	624.87**	0.65
PST	18.94 (9.78)	36.03 (9.8)	43.5 (6.17)	289.11**	0.46
PSDI	1.55 (0.44)	2.35 (0.46)	3 (0.41)	405.14**	0.55
Number of psychiatric diagnoses	1.38 (1.24)	1.74 (1.52)	2.61 (2)	26.6**	0.07
Impulsivity^a^	74.36 (5.21)	74.42 (6.01)	74.91 (5.77)	0.62	0
Acquired capability^b^	16.64 (6.86)	17.13 (6.72)	19.57 (6.5)	12.49**	0.04
Non-suicidal self-injury (%yes)	21.43	30.42	56.77	57.6**	0.2
Number of previous attempts	1.92 (1.54)	2.68 (2.65)	4.61 (7.78)	12.64**	0.04
Presence of suicidal ideation (%yes)	67.86	94.41	96.04	74.01**	0.32
Suicidal ideation intensity	11.88 (9.67)	18.6 (7.2)	21.31 (6.53)	56.02**	0.14
Presence of SRB (%yes)	50	65.38	79.87	32.94**	0.21
Number of SRB	0.75 (0.86)	1.04 (0.97)	1.62 (1.14)	34.53**	0.09
Current suicidal attempt					
Medical damage (%severe)	23.81	10.49	12.54	10.27**	0.08
Method^c^				20.98	0.10
Poisoning with solid or liquid substances	72.62	84.27	82.84		
Poisoning with gasses or vapors	1.19	2.45	0.99		
Stabbing	7.14	6.64	7.26		
Drowning	1.19	0	0		
Savings or strangulation	4.76	2.45	2.64		
Jumping from high buildings	3.57	0.70	2.31		
Other	9.52	3.50	3.96		

*Note:* The percentage of cases is displayed for dichotomous and categorical variables. Mean and standard deviation (between brackets) are displayed for continuous variables. The analysis of variance *F*-based tests (continuous variables) and *χ* ^2^ tests (dichotomous/categorical variables) were used as contrast test statistics. Effect size (ES) estimates were the *η* ^2^_partial_ for continuous variables and Cramer’s *V* for non-continuous ones.

Abbreviations: BSI, Brief Symptom Inventory; GSI, Global Severity Index; PSDI, Positive Symptom Distress Index; PST, positive symptoms total; SRB, suicide-related behavior.

a Total score of the Barratt Impulsiveness Scale (BIS-11).

b Total score from the Acquired Capability for Suicide Scale (ACSS).

c *χ* ^2^ test with *p* corrected using the Fisher’s exact test.

* *p* < .05.

** *p* < .01.

The generalized linear model for suicide outcome prediction revealed that the model with all the covariates (i.e., sociodemographic, clinical, and suicide-related ones) fitted better to data, as proven by its lower AIC, than the unconstrained model and model with sociodemographic covariates (see Table 4). The presence of suicidal ideation was featured by being a member of either the moderate-symptom class (*OR* = 7.80, *Z* = 5.60, *p* < .01) or the high-symptom class (*OR* = 8.70, *Z* = 5.07, *p* < .01), in comparison to the low-symptom class; and the acquired capability for suicide, with higher risk of ideation with higher ACSS scores (*OR* = 1.53, *Z* = 2.72, *p* < .01). The suicidal ideation intensity was associated with being a member of either the moderate-symptom class (*OR* = 1.53, *Z* = 8.25, *p* < .01) or the high-symptom class (*OR* = 1.66, *Z* = 9.29, *p* < .01), in comparison to the low-symptom class; the number of diagnoses (*OR* = 1.05, *Z* = 2.59, *p* < .05), the acquired capability for suicide (*OR* = 1.07, *Z* = 4.05, *p* < .01), and the number of previous suicide attempts (*OR* = 1.04, *Z* = 2.07, *p* < .05). In this regard, the higher all these clinical factors (i.e., higher number of diagnoses, higher acquired capability and higher number of previous suicide attempts), the more intense the suicidal ideation in last month. Regarding behavioral aspects, the presence of SRB was featured being a student in comparison to unemployed/retired participants (*OR* = 2.86, *Z* = 2.37, *p* < .05); the number of psychiatric diagnoses (*OR* = 1.35, *Z* = 2.49, *p* < .05), self-injury (*OR* = 2.26, *Z* = 3.73, *p* < .01), and the number of previous suicide attempts (*OR* = 4.71, *Z* = 4.51, *p* < .01). Again, an increased risk of SRB was associated with higher number of psychiatric diagnoses and previous suicide attempts, and the presence of self-injury. On the other hand, the number of SRB was positively linked with the number of psychiatric conditions (*OR* = 1.04, *Z* = 2.25, *p* < .01), the presence of self-injury (*OR* = 1.19, *Z* = 4.48, *p* < .01) and the number of previous suicide attempts (*OR* = 1.09, *Z* = 4.66, *p* < .01). In this case, the high-symptom class membership (in comparison to low-symptom class one) was positively associated with a higher number of SRB (*OR* = 1.26, *Z* = 3.87, *p* < .01). Finally, the severity of the current suicide attempt was associated with two covariates: the working status and the symptom class membership. More concretely, the active work status was associated with lower risk of severe medical injury derived from the current suicide attempt (*OR* = 0.41, *Z* = −3.06, *p* < .01). In terms of symptom class membership, both the moderate-symptom (*OR* = 0.40, *Z* = −2.74, *p* < .01) and high-symptom class (*OR* = 0.44, *Z* = −2.34, *p* < .05) memberships showed lower risk of severe medical injury derived from the current suicide attempt, in comparison to the low-symptom class.

**Table 4.** Covariates to explain suicide-related behavior outcomes in attempters

	Presence of ideation		Ideation intensity		Presence of suicide behavior		Number of suicide behaviors		Severity of current attempt	
	*OR* (*CI*_95_)	Z	*OR* (*CI*_95_)	Z	*OR* (*CI*_95_)	Z	*OR* (*CI*_95_)	Z	*OR* (*CI*_95_)	Z
(Intercept)	3.22 (1.17, 9.49)	2.20*	13.33 (11.73, 15.17)	39.66**	1.71 (0.83, 3.56)	1.45	1.81 (1.58, 2.09)	8.28**	0.28 (0.11, 0.64)	−2.92**
Sex (ref. male)	1.33 (0.70, 2.47)	0.89	1.01 (0.94, 1.09)	0.39	0.98 (0.65, 1.45)	−0.12	0.98 (0.91, 1.06)	−0.50	0.82 (0.5, 1.37)	−0.79
Age	0.98 (0.69, 1.38)	−0.14	1.02 (0.98, 1.06)	1.02	0.91 (0.74, 1.13)	−0.86	0.98 (0.94, 1.03)	−0.80	1.16 (0.88, 1.52)	1.07
Nationality (ref.: native-born)	1.01 (0.47, 2.31)	0.02	0.98 (0.91, 1.06)	−0.43	0.91 (0.58, 1.45)	−0.39	0.95 (0.87, 1.03)	−1.26	1.54 (0.87, 2.68)	1.51
Marital status (ref.: Married/coupled)	0.86 (0.45, 1.59)	−0.49	1 (0.93, 1.06)	−0.11	0.99 (0.68, 1.45)	−0.04	0.99 (0.92, 1.07)	−0.19	1.29 (0.80, 2.14)	1.03
Educational attainment (ref.: Primary)										
Secondary	0.77 (0.33, 1.71)	−0.62	0.97 (0.9, 1.05)	−0.68	0.71 (0.44, 1.12)	−1.45	0.94 (0.86, 1.03)	−1.31	1.03 (0.57, 1.92)	0.10
Tertiary	0.50 (0.20, 1.11)	−1.50	0.91 (0.83, 1)	−1.93	1.12 (0.65, 1.94)	0.42	1.03 (0.93, 1.14)	0.57	1.44 (0.74, 2.82)	1.07
Working status (ref.: Unemployed/retired)										
Active	1.46 (0.74, 2.95)	1.07	1.01 (0.95, 1.09)	0.40	1.29 (0.87, 1.93)	1.25	1.06 (0.98, 1.15)	1.54	0.41 (0.23, 0.72)	−3.06**
Student	2.42 (0.60, 16.41)	1.10	1.02 (0.91, 1.15)	0.39	2.86 (1.25, 7.24)	2.37*	1.12 (0.99, 1.28)	1.73	0.60 (0.22, 1.45)	−1.08
Symptom profile (ref.: Minimal symptoms)										
Moderate symptom	7.80 (3.85, 16.41)	5.60**	1.53 (1.38, 1.7)	8.25**	1.46 (0.86, 2.48)	1.41	1.1 (0.98, 1.23)	1.64	0.40 (0.21, 0.78)	−2.74**
High symptom	8.70 (3.86, 20.74)	5.07**	1.66 (1.49, 1.85)	9.29**	1.67 (0.94, 2.98)	1.74	1.26 (1.12, 1.42)	3.87**	0.44 (0.22, 0.88)	−2.34*
Number of psychiatric diagnoses	1.40 (0.95, 2.15)	1.62	1.05 (1.01, 1.08)	2.59*	1.35 (1.07, 1.71)	2.49*	1.04 (1.01, 1.08)	2.25*	0.95 (0.72, 1.23)	−0.39
Impulsivity^a^	1.33 (0.97, 1.84)	1.75	1 (0.96, 1.03)	−0.30	0.93 (0.77, 1.12)	−0.74	0.99 (0.96, 1.03)	−0.55	1.01 (0.8, 1.28)	0.11
Acquired capability^b^	1.53 (1.13, 2.08)	2.72**	1.07 (1.03, 1.1)	4.05**	1.09 (0.91, 1.32)	0.94	1.01 (0.97, 1.05)	0.55	0.96 (0.76, 1.22)	−0.30
Non-suicidal self-injury (ref.: No)	0.74 (0.37, 1.51)	−0.84	1 (0.93, 1.08)	0.07	2.26 (1.48, 3.5)	3.73**	1.19 (1.1, 1.29)	4.48**	1.49 (0.88, 2.52)	1.48
Number of previous suicide attempts	0.92 (0.72, 1.46)	−0.53	1.04 (1, 1.08)	2.07*	4.71 (2.5, 9.6)	4.51**	1.09 (1.04, 1.15)	4.66**	1.13 (0.92, 1.39)	1.25
Fit index										
AIC										
Unconstrained	433.38		5474.11		877.81		2072.13		538.43	
Sociodemographic	435.33		5427.95		851.41		2023.19		527.23	
Full	341.65		5044.69		724.71		1810.91		518.46	
*R* ^2^ (full model)	.35		.50		.33		.37		.13	

*Note:* Logistic binary regression was used for the presence of suicidal ideation and the presence of suicide-related behavior outcomes (reference category: absence for both outcomes), and for the severity of current attempt outcome (reference category: low severity). The suicidal ideation intensity and number of suicide-related behaviors outcomes were modeled using generalized linear modeling under gamma distribution. The unconstrained model did not include any covariate. The sociodemographic model comprised sociodemographic covariates (i.e., sex, age, marital status, education attainment). The full model comprised all the covariates, both sociodemographic and clinical.

Abbreviations: *AIC, Akaike information criterion; CI_95_, 95% confidence interval of the OR; OR, odds ratio; Z, Wald’s z-based statistic to test whether loading is significantly different from one.*

a Total score of the Barratt Impulsiveness Scale (BIS-11).

b Total score from the Acquired Capability for Suicide Scale (ACSS).

* *p* < .05.

** *p* < .01.

## Discussion

In line with Cuthbert (2022), a dimensional framework can help to advance the understanding of the etiopathology of disorders across the lifespan, as well as to optimize therapeutic choice. A dimensional standpoint may contribute to gain insight into the suicide risk dynamics of patients with a subclinical condition [32, 33]. Second, sociodemographic aspects, traumatic events, and even having skills and behavioral repertoires to cope with highly demanding situations can lead to feelings of distress, demoralization, and entrapment leading to suicidal behavior [12, 34, 35]. Third, the social context, such as the COVID-19 pandemic, directly impacts personal needs by introducing new sources of stress (e.g., uncertainty, economic instability, or interpersonal stress due to reduced social interactions) [36]. This may lead to an increased risk of suicidal behavior [37], despite a diagnosis of mental disorder could not be upheld due to lack of endorsement with several diagnostic criteria (e.g., temporal criterion).

We identified three groups of patients with a different profile of symptoms from a dimensional standpoint, interestingly according to the level of symptom intensity: the high symptom group (45.02% of participants) showed higher levels of symptoms across psychopathology dimensions; moderate symptom cluster (42.50%) and, low (or minimal) symptom cluster (12.48%). The anxiety-related and obsessive-compulsive domains of symptoms (i.e., anxiety, OCD, phobic, and somatization) were relevant to differentiate between cluster of participants, highlighting how complex social contexts, such as the pandemic, may contribute to anxiety symptom development and obsessive thought [38–40].

This study goes in line with previous findings, stressing the varying profile of symptoms that may exist among suicide attempters [19, 20, 41]. Divergencies between results from different studies may be probably due to the consideration of different risk factors to cluster patients (e.g., using specific symptom domains) and different life periods across studies. Most of the aforementioned studies failed to include key symptom domains that may be critical for suicide behavior (e.g., psychotic symptoms), as already described elsewhere [42–44]. In terms of life period, variability in suicide outcomes is clear, observing for instance, that old age people to have the highest mortality rate by suicide, in comparison with younger age groups [45, 46].

Regarding the clinical profile, we found that the members from the High-symptom cluster may be at greater risk of suicide outcomes, as they showed higher levels across the risk factors considered, except in the case of impulsivity (no between-group difference was observed). It is worth noting that our sample showed levels of impulsivity surpassing the cut-off point of clinical meaningfulness across the study group. Likewise, the attempters from the high-symptom profile also showed greater levels of suicide-related outcomes (i.e., presence and intensity of suicidal ideation, number of suicidal behaviors), as expected.

Of great interest is how lethal was the suicidal attempt of low-symptom cluster members (they represent 12.48% of the sample in study). Low-symptom members showed attempts with more severe medical outcomes than other members from the other profiles. This may be related to the fact that when the symptomatology is very high, the person may experience a greater discomfort and entrapment, but capacity for action may be limited due to the intensity of the psychopathological symptoms; while at lower symptomatic levels, feelings of desmoralization and entrapment may also be present and an increased risk of engaging in attempts featured by higher levels of success due to mild discomfort and higher capability to face the suicidal event [35, 47]. Moreover, it is more likely a low-symptom cluster attempter to be older and either be unemployed or retired. Although older people may be at higher risk of suicide and may show additional influence of other critical risk factors (e.g., multimorbidity, disability, widowhood), we speculate that pandemic-related economic and social hassles may be behind this increased risk of attempt lethality. The COVID-19 pandemic has put people at higher risk of poverty and economic insecurity [46, 48, 49].

This study has some limitations. First, our findings come from a cross-sectional study, so causal relationships cannot be tested. For that reason, the results should be cautiously interpreted in terms of variable association and future studies under a longitudinal perspective must provide further details to disentangle explanatory relationships. Likewise, some risk factors are missing, such as pharmacological treatment and prescribed medication. Moreover, biological measures were not incorporated in this study. For that reason, this study should be considered as a cornerstone to develop further studies providing specific evidence to go deeper into specific explanatory mechanisms. Moreover, data from this study were collected during the COVID-19 pandemic. Our results should therefore be framed on a critical period in human history, with subsequent difficulties to compare with other periods. Even though, they pave the way to better understand suicide risk in the post-pandemic times. Future studies adopting a symptom-based approach should cover a wider variety of risk factors (also biological), to make a more accurate picture of suicide attempt risk, and related behavior. Finally, our sample was selected intentionally; in other words, representativeness of our sample was not preserved, taking into account the difficulties to recruit patients from an emergency ward during the pandemic times.

To sum up, three symptomatic profiles were identified in a sample of suicide attempters, coming from a dimensional standpoint. Most of them showed a profile of moderate or high number of psychopathological symptoms. Attempters in the high symptom profile showed the highest risk and suicidal behavior results.

Relevant implications are derived from this article. First, suicide behavior may also occur in people with mild symptoms. Second, suicide attempt from people with low symptom profile showed a higher risk of a more severe attempt in terms of medical injury. This supports the importance of assessing suicidal behavior from a more dimensional view. It is proven that the risk of showing a greater injury due to suicidal behavior may not be directly associated with psychiatric diagnosis, nor with the high intensity of the symptoms. Suicidal behavior must be addressed from early or prodromal conditions, considering risk factors more related to the context, past experience and emotional states, and not only focusing on the mental diagnosis. Third, a broader assessment protocol becomes necessary to make an comprehensive picture of people at risk of suicide behavior. This protocol should collect data on the social, family, economic, and work spheres of the person, as well as mental health factors, even from very initial stages for the suicidal risk. Finally, it urges the wide implementation of prevention strategies, covering community populations (i.e., universal prevention), as the WHO recommends, to prevent suicidal behavior from people in which warning signals are highly difficult to be detected. This may definitely help save lives.
